# Capturing exposure in environmental health research: challenges and opportunities of different activity space models

**DOI:** 10.1186/s12942-018-0149-5

**Published:** 2018-07-28

**Authors:** Tiina E. Laatikainen, Kamyar Hasanzadeh, Marketta Kyttä

**Affiliations:** Department of Built Environment, Aalto University, PO Box 14100, 00076 Aalto, Finland

**Keywords:** Activity space, Exposure, Built environment, Wellbeing, Neighborhood, PPGIS

## Abstract

**Background:**

The built environment health promotion has attracted notable attention across a wide spectrum of health-related research over the past decade. However, the results about the contextual effects on health and PA are highly heterogeneous. The discrepancies between the results can potentially be partly explained by the diverse use of different spatial units of analysis in assessing individuals’ exposure to various environment characteristics. This study investigated whether different residential and activity space units of analysis yield distinct results regarding the association between the built environment and health. In addition, this study examines the challenges and opportunities of the different spatial units of analysis for environmental health-related research.

**Methods:**

Two common residential units of analysis and two novel activity space models were used to examine older adults’ wellbeing in relation to the built environment features in the Helsinki Metropolitan Area, Finland. An administrative unit, 500 m residential buffer, home range model and individualized residential exposure model were used to assess the associations between the built environment and wellbeing of respondent’s (n = 844).

**Results:**

All four different spatial units of analysis yield distinct results regarding the associations between the built environment characteristics and wellbeing. A positive association between green space and health was found only when exposure was assessed with individualized residential exposure model. Walkability index and the length of pedestrian and bicycle roads were found to positively correlate with perceived wellbeing measures only with a home range model. Additionally, all units of analysis differed from each other in terms of size, shape, and how they capture different contextual measures.

**Conclusions:**

The results show that different spatial units of analysis result in considerably different measurements of built environment. In turn, the differences derived from the use of different spatial units seem to considerably affect the associations between environment characteristics and wellbeing measures. Although it is not easy to argue about the correctness of these measurements, what is evident is that they can reveal different wellbeing outcomes. While some methods are especially usable to determine the availability of environmental opportunities that promote active travel and the related health outcomes, others can provide us with insight into the mechanisms how the actual exposure to green structure can enhance wellbeing.

## Background

Built environment health promotion has attracted notable attention across a wide spectrum of health-related research over the past decade [1–4]. Several built environment features, such as connectivity, density, walkability, and mixed land use, have been found to be positively associated with both perceived and objectively measured health, physical activity (PA), and active mobility [5–9]. Furthermore, research has shown that green areas have positive wellbeing effects and exposure to natural settings decreases stress and increases positive affect [10–12]. Studies have also found links between walkable neighborhoods and decreases in prevalence of overweight, obesity, and incidence of diabetes [13, 14]. In addition, neighborhood walkability has been linked to increases in cardiorespiratory fitness [15].

However, the results of the prior research about the contextual effects on health and PA are highly heterogeneous [16–18]. The discrepancies between the results can potentially be explained by the diverse use of different spatial units of analysis in assessing individuals’ exposure to various environment characteristics [19–23]. The vast majority of previous studies has concentrated on analyzing the built environment features around individuals’ residences or “neighborhoods” that have been delineated through administrative units (e.g., census tract, postal code areas) or residential or workplace buffers with varying radii and buffering methods [22, 24]. These units have been a popular way of defining the spatial extent of individuals’ exposure to different environmental features, mostly due to their availability and ease of use.

Network and “sausage” buffers that create polygons around individuals’ residences based on the street network have become commonly applied spatial units of analysis to define individuals’ neighborhoods in public health research [19]. While it is evident that various network buffers are superior to simple administrative units or circular buffers, these units are also still unable to characterize the space within which people actually move around [25]. Despite the advances in the buffer-based units of analysis, these approaches account only for the built environment characteristics around individuals’ residences and neglect the spatial realities of where, as well as the temporal aspect of when and how long, individuals are moving around [20, 26]. Due to the static nature of these units of analysis, it is assumed that individuals are exposed solely to the environment around their residency and, thus, manage to capture only a hypothetical individual exposure. According to Kwan [18], this “uncertain geographic context problem (UGCoP)” is one of the reasons why research findings concerning the effects of the built environment on health have been found to be inconsistent.

In their literature review, Leal and Chaix [25] found that 90% of the studies examining the associations between built environment and cardiometabolic risk factors focused their analysis solely on residential environments [25]. The problematic nature of most of these kinds of studies, where the research is limited to static neighborhoods, has also been highlighted within the new mobilities paradigm [27, 28]. The “mobility turn” in social sciences underlines the essential role of person-based and dynamic analysis and stresses a move forward from static spatial approaches [29]. Similarly, studies examining the associations between built environment and health have noted the complexity of defining individuals’ spatial exposure and “local” neighborhoods [30].

Recent research has been taking steps toward more dynamic and person-based units of analysis to define the spatiotemporal extents of individuals’ neighborhoods and spatial exposure by capturing the notions of activity space [21, 26, 31–33]. These studies account for individuals’ actions and mobility behaviors within and exterior to their residences and local neighborhoods to overcome the uncertainty of the geographic context [18, 32]. The studies have used GPS tracking devices as well as online participatory mapping methods to collect data about the spatial extent of individual behavior [20, 26, 32, 33].

Hasanzadeh et al. [33] have developed a versatile model of activity spaces using an individual-based delineation of places of everyday activities. In their study, Hasanzadeh et al. [33] created an individual-based definition of activity spaces that are dynamic in their boundaries. Later Hasanzadeh and colleagues [26] introduced a more spatially sensitive model of individual activity spaces using a notion of place exposure. This individualized residential exposure model (IREM) is based on the understanding that the influencing context is more than a delineated area around an individual’s place of residence and everyday activity places. According to Hasanzadeh et al. [26], a more refined picture of activity spaces can be achieved through an estimation of place exposure and its variation throughout an individual’s activity space. In their study, Kestens et al. [32] compared activity spaces delineated from GPS tracking with activity spaces delineated from an online participatory mapping questionnaire on regular destinations and concluded that self-reported destinations provided a representative picture of study participants’ spatial realities of where they move around.

Despite the notions that the research on the built environment impacts on health and PA yields varying results, only a few studies have compared whether the association between built environment characteristics and health outcomes differs when using various spatial units of analysis in capturing the context. Zenk et al. [22] found evidence that environmental features were related to participants’ weight-related behaviors when using modeled activity spaces as units of analysis, but the environmental features of mere residential neighborhoods were not. In their study, Howell et al. [20] assessed how PA and walkability associations vary when different spatial measures were used and reported stronger associations between PA and walkability with activity spaces rather than with the simple home neighborhood unit of analysis. In their international study, Frank et al. [19] compared different buffering methods and concluded that the values of built environment measures differed significantly between detailed, detailed-trimmed, and sausage buffers. Holliday et al. [31] reported that simple residential buffers are an ill-fitting unit of analysis solution without assessing prior to each study if the simple residential neighborhood is an appropriate exposure area to study the health behavior in consideration. In a recent study, Zhao et al. [23] found that activity space measured with either standard deviational ellipse, minimum convex polygon or road network buffer influenced if and how an environmental variable affected obesity. According to Kestens et al. [34], activity spaces created both using GPS and map-based questionnaires can provide a way to overcome the contextual problems and improve our understanding about the mechanisms that connect place to health.

In this study, we seek to build on the previous, yet rather limited, research that have compared whether the associations between the built environment measures and health differ when individual exposure is assessed with various spatial units of analysis including the latest person-based models [19, 31, 32]. The goal of this study is to investigate whether different spatial units of analysis yield distinct results regarding the association between the built environment and health. This study also examines the challenges and opportunities of the different spatial units of analysis for environmental health-related research.

We applied two common residential units of analysis and two novel activity space models to examine older adults’ perceived wellbeing in relation to the built environment features in the Helsinki Metropolitan Area (HMA), Finland. In detail, we investigated whether the associations between commonly used built environment measures and perceived wellbeing outcomes differ when using an administrative unit, residential buffer, home range model [33], or IREM [26] for assessing the individual environmental exposure. We also compared how the four different models match with activity spaces delineated from GPS activity spaces collected from a subsample of the study participants. The GPS tracking offers data about human mobility behavior free of self-report bias [34] and, thus, offers a possibility to study the challenges and opportunities of the different spatial units of analysis based on participatory mapping in comparison to GPS activity spaces.

## Methods

### Study area and participants

A random sample of 5000 residents of HMA aged between 55 and 75 years received an invitation letter by mail in September 2015 asking them to participate in an online survey. A total of 1139 full or partial responses were received, and after removing incomplete responses, 844 were taken for further analysis. Participants consisted of 447 women and 331 men with a mean age of 64.3 (SD 5.52). The data showed general consistency on most sociodemographic variables within the study region (Table 1). A raffle for five hundred euro gift cards was arranged between all participants. Aalto University’s Research Ethics Board approved the study.

**Table 1:** Explanations of the abbreviations for the variables in the equation

Abbreviations	Variables
WB	Wellbeing measure
Gr	Greenness
LUM	Land use mix
W	Walkability
PC	Pedestrian/cycling routes
S	Size^a^
I	Income
AG	Age
G	Gender
EDU	Education
RT	Retirement status

^a^Not included for buffer and administrative unit models

The data were collected using a place-based mapping method, public participation GIS (PPGIS), which combines internet maps with traditional questionnaires [35]. PPGIS has offered convenient tools for previous studies investigating human behavior in a context-sensitive way [36–39]. Localization of human experiences and behavioral patterns by participatory mapping tools attaches them to specific physical environmental context [40]. Thus, human behavior and experiences receive geographic coordinates, which allows simultaneous GIS-based analysis of human behavior in relation to the physical environment [35]. In the survey, the respondents used an online interface to mark their everyday errand points (EEPs) on a map. In addition, the respondents indicated which transport mode they used and how frequently they accessed the places. The survey was created with Maptionnaire^®^ tool. With the place-based mapping method, we were able to study older adults’ spatial behavior context-sensitively by asking respondents to think about their typical week and mark on the map all sorts of everyday places they visit during the week. Simultaneously, the respondents’ personal background characteristics were studied by asking them to answer several questions related to their sociodemographic background.

Additional GPS data were collected to compare and validate how the different models of activity spaces match with the GPS tracks collected from a subsample of participants. A subsample of 100 participants of the PPGIS survey were selected, and an invitation to participate in additional GPS data collection was sent to participants’ home addresses in September 2016. The participants were offered a 50 euro gift card for their participation. Twenty-nine participants used GPS devices with built-in accelerometers (Sensedoc ™ 2.0; MAX-M8 Global Navigation Satellite System receiver from u-blox, 2 s epoch, Tri-axial accelerometer, 50 Hz) for eight consecutive days. The participants also kept travel diaries during the same eight-day period. Those participants with valid data for GPS were included in the analysis, thus leaving a final sample size of 18 individuals. Excluded users were people who reported having an unusual week, traveled extensively outside home surroundings during the study, or had problems using the device. The excluded users did not show any significant differences to the rest of users in terms of socio-demographics.

### Activity space models

**Fig. 1:**
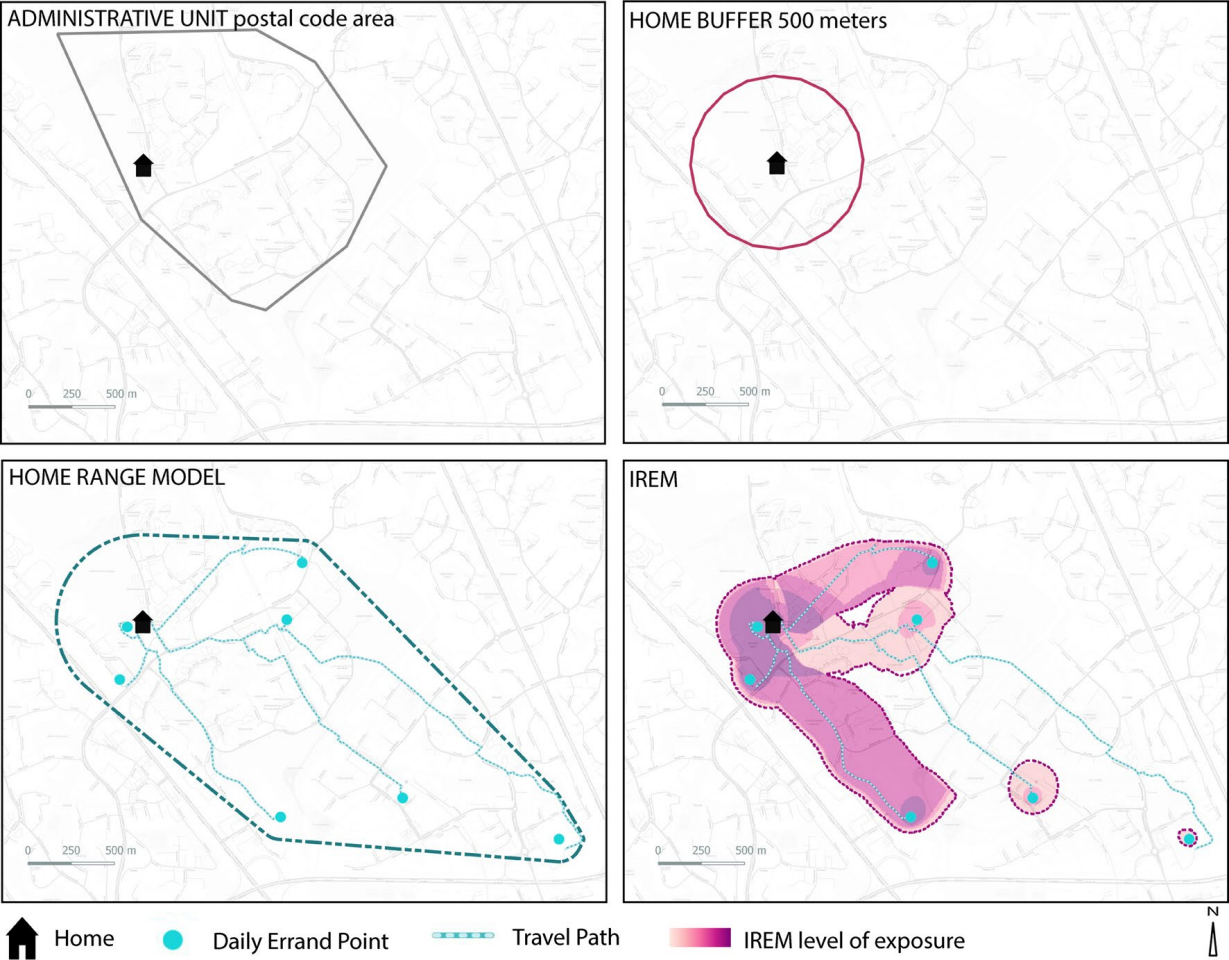
The four different ways respondents’ activity spaces were modeled

We implemented and subsequently compared four different spatial units of analysis. While all four units of analysis are individual-based, they differ significantly in terms of their complexity (Fig. 1). All four units of analysis will be referred to here as models of activity spaces, regardless of the complexity and the way they have been delineated. The first models are technically simple while the other two are more individual-specific and therefore come with higher technical complexities.

The first model, administrative boundary, is based on the postal areas and is determined for each individual based on their place of domicile. The second model is a circular buffer around each individual’s home. This buffer was implemented using a static radius of 500 m, which is adopted as a commonly used distance in the literature [33]. The third model used, home range, is an individual-specific boundary method, which was first introduced by Hasanzadeh et al. [33]. Following the criteria suggested in the study, we listed all EEPs based on their distance from the participant’s home location. The Jenk’s optimization method revealed 4 km as the suitable home range distance for the data set [33, 41, 42]. This distance is based on the first natural break value including at least 80% of EEPs marked by the participants. It should be noted that the optimum number of classes for the Jenk’s algorithm was determined using Goodness of Variance Fit (GVF) [42]. In the next step, a convex hull was applied to enclose all EEPs as well as the home point. However, prior to the implementation of convex hulls, buffers were applied to each point marked by the participants. Accordingly, buffers with distances 500 and 140 m were applied to the home locations and EEPs, respectively. According to Hasanzadeh et al. [33], 500 m is the most frequently used distance for defining immediate neighborhoods in literature, and 140 m is identified as a suitable estimation of activity cluster sizes in a data set collected from the same area as the current study. The later distance was calculated in the study as the average diameter of the spatial clusters formed by the aggregate of EEP markings [33].

The fourth model, IREM, is an exposure-based model of activity spaces [26]. Following the IREM criteria, we estimated the level of place exposure for each respondent throughout individual activity spaces using information on home location, visited places, frequency of visits, travel paths, and use of travel modes. In the IREM approach, exposure is expressed by assigning weights for places visited in terms of reported visit times per month with highest frequency of visits assigned to the home location. In addition, IREM estimates the level of exposure by taking into account the travel behavior of each individual. In IREM, the weight assigned to each travel path consists of the geometric average of weights at the origin and destination points and the average speed of the reported travel mode [26]. As an example, an individual who reported a higher frequency of use for a certain path with a non-motorized transportation mode is assumed to have higher exposure to his or her surroundings along their trip route compared to a less-frequently traveling individual who uses motorized transportation modes.

In the last step of creating IREM, an inverse distance function was applied to produce a raster representing the activity space of each individual [26]. The raster is made of square pixels with dimensions 25 m × 25 m, each containing a value as the estimation of exposure magnitude in its corresponding location. The exposure values are normalized using a sigmoid function with 0 as the minimum exposure and no upper limit defined for highest exposures [26]. The boundary of IREM was defined by the polygon encapsulating the high exposure areas. In this study, high exposure areas were identified as places with exposure values of more than 50% of the individual maximum.

Figure 1 shows an example of all four activity spaces modeled. It should be noted that we did not have any information about the actual travel path in the data set for the home range and IREM modeling. Therefore, the shortest path between each participant’s home location and their EEPs was found using the Network Analyst toolbox of ArcGIS 10.5. The transportation mode indicated by the participant for visiting each specific EEP was taken into consideration while choosing the shortest path.

### Dependent variables

Four different perceived wellbeing measures were used to test the associations between health and built environment measures. In the survey, the respondents were asked, how would they describe their (1) overall health situation, (2) ability to function, (3) quality of life, and (4) state of happiness at the moment. Respondents were asked to evaluate these four perceived wellbeing measures using a five-point Likert scale that ranged from very bad to very good. According to an extensive review by Kerr and colleagues [3] the evidence is building to suggest that older adults’ physical health and functioning is connected to built environment factors. There is also evidence that perceived as well as objectively measured human health is in link with the physical environment characteristics, such as green spaces [10, 12, 43]. The overall health situation and quality of life as well as the happiness measure have been used in previous studies about the contextual effects on perceived health and on gross national happiness [9, 44]. The functional ability was included as an additional measure targeting the specific older age group of the study [3].

### Independent variables

The built environment features have been found to be positively associated with health in many studies across the globe [5–9]. Thus, we used five different built environment measures to test their possible associations with perceived health of individuals. We measured amount of green spaces [7, 10, 11], the size of the modeled activity space [22, 26], land-use mix (LUM) [8] and walkability [2, 7] as walkability index and the length of pedestrian and cycling routes. The five measures were derived in GIS for administrative unit, 500 m residential buffer, home range model and IREM.

#### Greenness

This is a measure showing the amount of green areas within each individual activity space. For the three boundary approaches, namely administrative boundary, buffer, and home range, this was simply calculated as the percentage of land covered by green areas. For IREM, this was defined as the percentage of green exposure, and it was operationalized as the ratio of exposure to green areas to the total exposure within the activity space. Green exposures were determined as the value of exposure to the pixel identified as green, as estimated by IREM.

#### Size of activity space

This is a geometric measure capturing the total surface of the activity space areas. For IREM, the high-exposure polygon was used to measure the surface.

#### Land-use mix

Higher mix of land use has been shown to enhance PA because it provides versatility to the built environment and provides a variety of destinations closer to each other [7]. The LUM measure considered four land-use types: residential, commercial, traffic, and green space. Previous studies have also considered entertainment, office, and institutional land uses for the mix measure [45, 46]. We adopted these particular land-use categories for the LUM measure for two reasons: available data sets and they provide the best possible correspondence to the actual built environment in the study area. The formula used to calculate the LUM was modified from the formula used by Frank et al. [46]:

$$H = {{ - 1 \left( {\mathop \sum \limits_{i = 1}^{n} pi*\ln \left( {pi} \right)} \right)} \mathord{\left/ {\vphantom {{ - 1 \left( {\mathop \sum \limits_{i = 1}^{n} pi*\ln \left( {pi} \right)} \right)} {\ln \left( n \right)}}} \right. \kern-0pt} {\ln \left( n \right)}}$$

where H is the LUM score, *pi* is the proportion of land use *i* among all land-use classes, and *n* is the number of land-use types. The information concerning land use, including green areas, was calculated from the CORINE dataset which is a raster dataset that provides information on Finnish land cover and land use on 2012. The data of CORINE has been produced as a part of the European Gioland 2012 project by Finnish Environment Institute (SYKE).

#### Walkability

Walkability was assessed according to the walkability index [45]. Walkability index was calculated as the sum of the z-scores of the four urban form measures [(2 × z-intersection density) + (z-net residential density) + (z-commercial floor area ratio) + (z-land-use mix)]. The measures were drawn from CORINE as well as from Digiroad that is an open road and traffic dataset provided by the Finnish Transport Agency.

#### Length of pedestrian/cycling routes

This measure was calculated as the total length of pedestrian and cycling routes in meters per square meter of the area of the spatial unit of analysis. For IREM, the high exposure polygon was used for the measurement. The pedestrian and bicycle roads were drawn from Open Street Map which is open geospatial data produced by a community of mappers. The data of OSM is fully open and licensed under the Open Data Commons Open Database License (ODbL) by the OpenStreetMap Foundation (OSMF).

### Statistical analyses

The statistical analysis of the different activity space models had three main objectives. The first was to compare the contextual variables obtained with different activity space models in order to examine whether they are significantly different. Paired sample *t* tests were utilized to examine whether significant differences exist between contextual variables calculated using different activity space models. The significance of comparison results is adjusted for type I error using Bonferroni correction.

Second, we compared how the four activity space models match with the GPS tracks collected from a subsample of participants. To do so, we overlaid the GPS tracks with each model and calculated the percentage of overlap before applying the 4-km locality threshold and then after it.

Third, we conducted four independent regression analyses using multivariate linear regression. Each analysis took all the five activity space-based built environment measures as the independent variables and one of the four wellbeing measures as the dependent variable. All regression models were controlled for gender, age, education level, income, and retirement state. The purpose was to analyze the effect of activity space-based built environment measures on participants’ perceived wellbeing and see how the choice of activity space model can affect the significance of observed associations. The model is as follows:

$${\text{WB}} = \upbeta_{0} + \, \upbeta_{1} {\text{Gr}} + \upbeta_{2} {\text{LUM}} + \upbeta_{3} {\text{W}} + \upbeta_{4} {\text{PC}} + \left( {\upbeta_{5} {\text{S}}} \right) + \upbeta_{6} {\text{I}} + \upbeta_{7} {\text{AG}} + \upbeta_{8} {\text{G}} + \upbeta_{9} {\text{EDU}} + \upbeta_{10} {\text{RT}} + \upvarepsilon$$

The independent variables of the model are explained in Table 1.

## Results

### Comparative analysis of activity space extents and measures

**Fig. 2:**
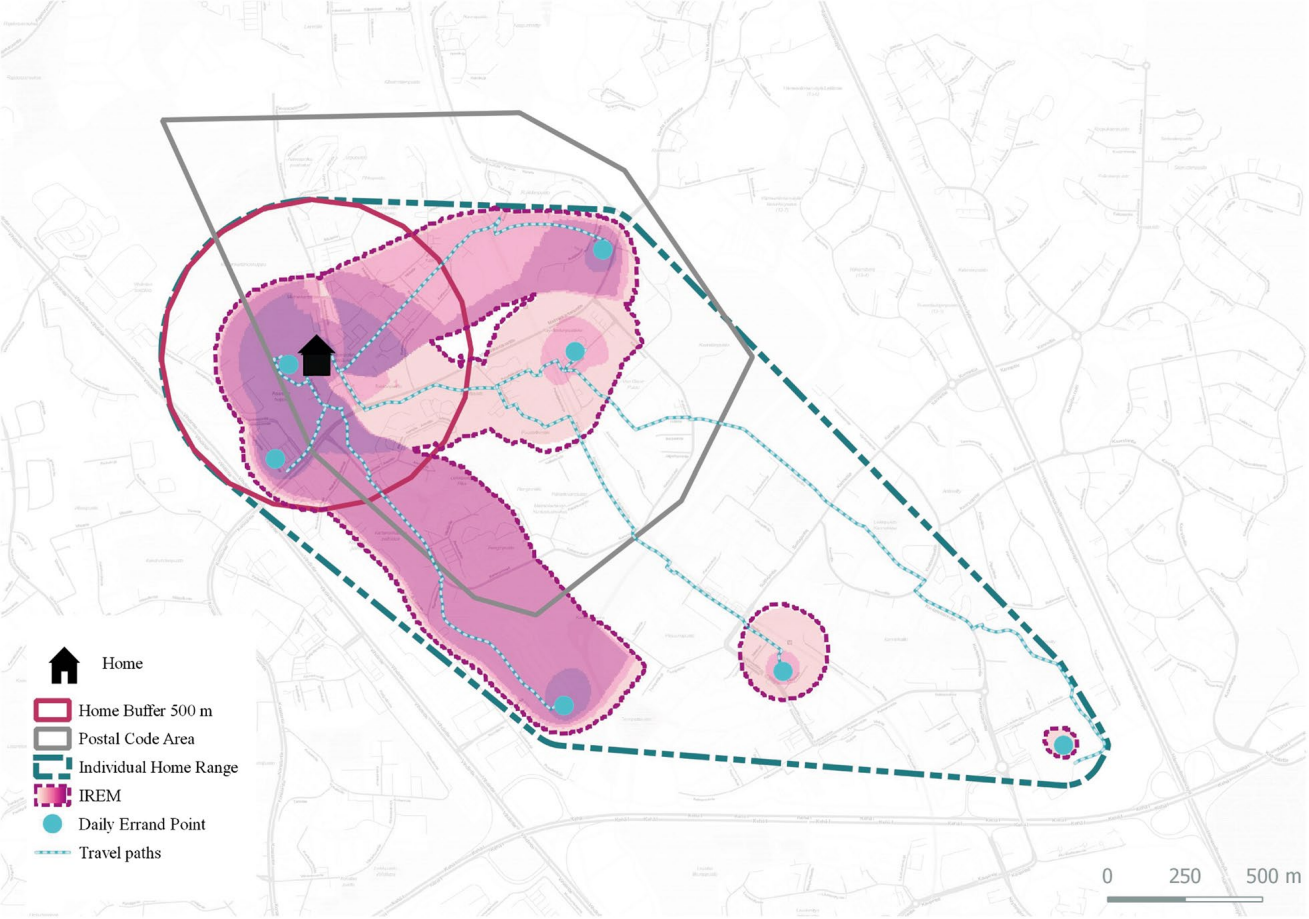
The spatial overlap of all four activity space models used in this study

As expected, the four models vary significantly in size and shape (Table 2). The results show that the home range model has the most overall overlap with other methods, whereas the buffer approach shares the least overlaps among the methods (Fig. 2). Similarly, as Table 3 shows, the home range model has the highest level of consistency with GPS activity space. The home range model covered over half of the total GPS activity space, and the spatial overlap was 40% for IREM. Administrative unit and buffer covered a little less, with mean overlap of 38% for the former and 35% for the latter of the whole GPS activity space. When a 4-km cutoff distance to the GPS activity space was considered, the home range model covered nearly 80% and IREM two-thirds of the GPS activity space. The buffer approach shows the lowest match rate with GPS activity space with an average overlap of around 55% within the 4-km locality threshold (Table 3).

**Table 2:** The percentage overlap of the four activity space models (n = 844)

	Administrative unit	Home buffer 500 m	Individual home range	IREM
Administrative unit	–	66.6	46.5	44.4
Home buffer 500 m	26.5	–	22.4	37.3
Individual home range	78.7	100	–	100
IREM	48.2	67.3	40.4	–

**Table 3:** The spatial overlap of the four activity space models with GPS activity spaces collected from a subsample of the study participants

Method	Mean overlap % (overall)	Mean overlap % within locality threshold (4 km)
Administrative unit	38	59
Home buffer (500 m)	35	55
Individual home range	56	79
IREM	40	65

Next, we performed paired sample *t* tests to examine whether significant differences are evident in the activity-space-based contextual measures obtained from different models. Table 4 summarizes the significance of paired sample *t* tests for the contextual variables between the activity space models for the 844 participants. As shown, significant differences exist for most of the pairs of the activity space measures. However, there are also pairs of activity space models that do not yield statistically significantly different measurement outcomes. Measurement of LUM does not significantly vary between any pairs of activity space models. Further, the value for walkability index does not significantly vary between some pairs of activity space models. The walkability index calculated using IREM appears to be significantly different from the one obtained using the home range model. Nevertheless, it does not seem to be significantly different from values obtained from the buffer and administrative boundary activity space model.

**Table 4:** The significance of paired sample *t* tests for the contextual variables between the four activity space models

	Admin. unit	Buffer	Home range	IREM
Greenness				
Admin		*<* *0.001*	*<* *0.001*	*<* *0.001*
Buffer	*<* *0.001*		*<* *0.001*	*<* *0.001*
HR	*<* *0.001*	*<* *0.001*		*<* *0.001*
IREM	*<* *0.001*	*<* *0.001*	*<* *0.001*	
Size of activity space				
Admin		*<* *0.001*	*<* *0.001*	*<* *0.001*
Buffer	*<* *0.001*		*<* *0.001*	*<* *0.001*
HR	*<* *0.001*	*<* *0.001*		*<* *0.001*
IREM	*<* *0.001*	*<* *0.001*	*<* *0.001*	
Land use mix				
Admin		0.797	0.801	0.803
Buffer	0.797		1.000	1.000
HR	0.801	1.000		1.000
IREM	0.803	1.000	1.000	
Walkability index				
Admin		*<* *0.001*	*<* *0.001*	0.803
Buffer	*<* *0.001*		1.000	1.000
HR	*<* *0.001*	1.000		*<* *0.001*
IREM	0.803	1.000	*<* *0.001*	
Pedestrian roads				
Admin		*<* *0.001*	*<* *0.001*	*<* *0.001*
Buffer	*<* *0.001*		*<* *0.001*	*0.002*
HR	*<* *0.001*	*<* *0.001*		*<* *0.001*
IREM	*<* *0.001*	*0.002*	*<* *0.001*	

A significance level of 0.05 (with adjustment for multiple comparison *p* < 0.008) is used to judge whether two measures are significantly different. The italicized values are statistically significant

### Regression analysis of health measures

In this section, we report the results of the regression analysis that explored how the contextual variables derived with different activity space models are associated with different aspects of health. To demonstrate whether the choice of activity space model influences the results, we compare the regression analysis results based on the significant associations found using the four activity space models.

Table 5 summarizes the results of the regression analysis based on different activity space models. The coefficients found to be statistically significant (Table 5) vary greatly between different activity space models. Some of the associations found via different activity space models contradict with each other. Greenness was found to be positively associated with several domains of health when using IREM as the activity space model; however, an opposite trend (significant negative associations) was found when home buffer and, in one case, when administrative boundary were used as activity space models.

**Table 5:** The association of contextual variables derived from different activity space models with different aspects of health

	Greenness				Land use mix				Walkability				P/C routes				Size			
	Administrative unit	Buffer	Home range	IREM	Administrative unit	Buffer	Home range	IREM	Administrative unit	Buffer	Home range	IREM	Administrative unit	Buffer	Home range	IREM	Administrative unit	Buffer	Home range	IREM
Health	*–* *0.25*	–	–	*0.01*	–	–	–	–	–	–	*0.10*	–	*0.11*	–	*0.13*	–	*NA*	*NA*	–	*0.10*
Func	*–* *0.03*	–	–	*0.09*	*0.15*	–	–	*0.10*	–	–	–	–	*0.08*	–	*0.09*	–	*NA*	*NA*	–	–
QOL	*–* *0.17*	*0.08*	–	*0.09*	–	–	–	–	–	–	*0.12*	–	–	–	*0.19*	–	*NA*	*NA*	–	*0.14*
Happiness	*–* *0.1*	–	–	–	–	–	–	–	*0.18*	*0.09*	*0.10*	–	–	–	*0.19*	–	*NA*	*NA*	–	*0.14*

*AU* administrative unit, *BF* buffer, *HR* home range, *IR* IREM

Only the coefficients significant at level *p* < 0.05 are shown in the table and those that are significant at level *p* < 0.01 are underlined

The size of activity space appears to be positively associated with several aspects of health when measured via IREM. It is noteworthy that the size of postal areas is based on administrative preferences, and circular buffers have arbitrary and static areas. Accordingly, the notion of size when using these two approaches was deemed irrelevant and, therefore, left out of the regression analysis.

Positive associations were found between the length of pedestrian and cycling routes within an activity space and walkability index of an activity space with different aspects of wellbeing. These associations were best found using the home range model. LUM was not found to be statistically significantly associated with wellbeing measures in most cases.

## Discussion

The aim of this study was to examine whether the associations between the built environment measures and health differ when individual exposure is assessed with several different spatial units of analysis. Earlier studies have concluded that the mixed results of the impact of the built environment on health can be at least partly due to the diverse use of different spatial units of analysis aiming to capture the individual spatial exposure [18–20, 23, 31]. There are only a few studies that have truly intended to study and overcome the contextual problems related to place exposure [23, 32].

We compared two common residential and two novel activity space models as units of analysis to investigate whether differences exist in the associations between built environment features and perceived individual wellbeing outcomes. According to the results, all units of analysis differed from each other in terms of size, shape, and how they capture different contextual measures. These findings support the existence of the uncertain geographic context problem when examining the association between the built environment and human health [18]. In addition, we found that the associations between commonly used built environment measures and perceived wellbeing outcomes differed when using an administrative unit, residential buffer, home range model, and IREM for assessing the individual environmental exposure.

The four studied units of analysis—administrative unit, 500-m residential buffer, home range model [33], and IREM [26]—varied significantly in their size and shape. Thus, all four models are very distinct from each other. In general, administrative units and residential buffers manage to capture only a hypothetical individual exposure as these methods presume that individuals are exposed solely to the environment around their residency or within administrative boundaries. Both are static models and do not capture the dynamic nature of everyday human behavior [23, 26]. The administrative unit covered less than half and buffer only around one-third of the area that the home range model and IREM captured, which was shown when the different models were overlapped with each other. The uncertain geographic context problem is evident for these kinds of static spatial units of analysis that cannot capture individuals’ true daily activities [23]. In contrast, the dynamic and people-based spatial models that use place-based data collected with online participatory mapping methods manage to capture the notions of activity spaces in a more individualized way [21, 26, 32, 33]. The home range model had the most overall overlap with other models. The home range model is an individual-specific boundary method created to capture individual activity spaces. Thus, the home range model captures the complete geographic area where individuals report themselves moving around. However, the individual home range model fails to capture varying levels of place exposure as it does not account for any temporal aspects. In contrast, IREM captures the areas where an individual is exposed to the physical environment according to the reported activities, frequency of visitations, and modes of transport used. Thus, the areal coverage of IREM is smaller than in home range models, but its capability to capture precise individual exposure is superior to any other models studied here.

Comparison of the four different spatial units of analysis with activity spaces delineated from GPS tracking demonstrated parallel results. The overall mean spatial overlap between the four studied models and a GPS activity space was highest for the novel people-based models. The home range model covered nearly 80% of the GPS activity space when a 4-km cutoff distance was applied to the GPS activity space. In comparison, the buffer covered only half of the same space. These results are in line with a study by Kestens et al. [32] where they found that both GPS and map-based questionnaires provide novel and suitable ways to collect daily mobility data and improve the exposure assessment of context-specific health research. Yet, it is noteworthy that GPS data collections are highly costly and time consuming. Thus, online map-based questionnaires can provide cost-efficient solutions for health research to delineate individual activity spaces in studies where context plays a significant role.

The differences of the contextual measures obtained from different models reassert the results of previous studies [20, 23]. We found significant differences in most built environment measures calculated using different activity space models. The amount of green space, the size of the activity space, and the length of pedestrian and bicycle roads differed significantly between all the activity space models. In their study, Zhao et al. [23] also found evidence that built environment measures differed between several activity space models. Nevertheless, there were a few exceptions in our results. LUM did not significantly vary between any pairs of activity space models. This might be because of the land use characteristics of the study area, which were rather mixed within the whole HMA in general. The conventional single-use zoning does not exist similarly in the study area when compared to North America where LUM has been commonly used as a well-fitting measure in environmental health studies. Additionally, a recent large international comparison found LUM not being related to PA [7]. Further, walkability index did not significantly differ between the static models and the activity space models. This might be related to the residential self-selection bias [47], where people who prefer walking may seek out to both live in and move around in walkable areas. In this case, respondents who prefer walking and active lifestyles seek to live in areas that are highly walkable (captured by administrative unit and home buffer) and move around in highly walkable areas (captured by individual home range model and IREM).

The associations between commonly used built environment measures and perceived wellbeing outcomes differed when using an administrative unit, residential buffer, home range model, and IREM for assessing the individual environmental exposure. The association between green space, LUM, walkability index, length of pedestrian and cycling routes, and wellbeing was assessed with all four different models. Additionally, the association between the size of the exposure model and health was assessed.

Only two very distinct models showed any results between the green space and health. When the individual exposure was assessed with IREM, a positive association between green space and health was found. For the administrative unit, a negative association was found. Thus, the amount of green space was found positively associated with respondents’ perceived overall health, functional capability, and quality of life when the exposure was assessed with a unit of analysis that accounts for the true individual exposure and activities undertaken in a certain place. On the other hand, the amount of green space was found negatively associated with respondents’ perceived wellbeing measures when administrative unit was used as a spatial unit of analysis. Thus, higher green area proportions around the residency decreased the perceived wellbeing. This is a rather contradictory result compared to previous studies on nature, green space and health [48, 49]. These results suggest that the true exposure to green instead of availability of green spaces are important to wellbeing. Thus, the accessibility, quality, and desirability instead of quantity of green spaces could be the focal aspects in planning healthy cities [50]. Additionally, these results can be explained by the fact that residential areas with vast green land uses in HMA are mostly fringe suburban areas surrounded by large forests and are highly car-oriented and less connected than the core urban areas. Thus, it might be that the quality of certain green areas instead of quantity of green space around one’s home matters to the wellbeing of the people living in HMA [9].

LUM was positively associated with perceived functional capability when measured with administrative unit but not with any other unit of analysis or any other perceived health measure. LUM alone is perhaps not an applicable measure because its association with health, PA, and neighborhood satisfaction has shown no significant associations [7, 51]. Once LUM is combined with other urban structural measures, such as building and residential density, it forms a more applicable measure to capture the true mixed character of the urban areas, which is not limited only to the horizontal mix of land uses but extends also to the mix of vertical urban space [52].

Walkability index and the length of pedestrian and bicycle roads were found to have a positive correlation with most of the perceived wellbeing measures when studied with the home range model as a unit of analysis. Thus, the more walkable the complete geographical area of the activity space is, the healthier, functionally more capable, and happy the respondents perceive themselves. Interestingly, no association was found between walkability index and perceived health when IREM was used as a unit of analysis. This discrepancy between the two activity space measures warrants further investigation because the results suggest that true exposure to walkable environments does not associate with health but that it is the availability and supply of highly walkable environments that associates with perceived health. Future studies investigating how walkability potential of activity spaces affect PA behavior of individuals compared to the exposure to walkable physical environments would continue to advance the understanding of the built environment relationship with health behavior.

By using the novel activity space models, future research could capture the various health promotive aspects of (urban) environments. The health impacts of the environment are complex, as the environment can affect all the physical, mental, and social health and wellbeing of individuals as well as the whole society [2, 6, 53, 54]. However, different characteristics of the environment can support mental health [10] compared to those that support physical health [7]. Exposure to green areas have been shown to reduce stress levels, and at the same time, well-connected, walkable, and dense urban environments support PA. These characteristics of the physical environment and their association to individuals’ wellbeing can be captured with the different spatial modelling techniques.

In line with previous research, the size of activity space was found to be positively associated with different aspects of wellbeing. This is related to the actual exposure measurement because the size shows statistically significant associations with wellbeing only when it is measured with IREM. Given the more concentrated characteristic of IREM, and the fact that the use of active travel modes contribute positively to the exposure to the surrounding areas, this association could be attributed to the greater extent of active travel. Active mobility is an important source of daily PA for many people and, thus, may improve general health [55–59]. A similar conclusion cannot be drawn from less concentrated activity space models, such as home range, because a bigger size of activity space in these models is more likely to be associated with car use, which is found to impede PA [60].

This study aimed at investigating the challenges and opportunities of different spatial units of analysis—not determining which one should be used. Our results show that different models of activity spaces result in considerably different measurements of built environment. In turn, the differences derived from the use of different spatial units seem to considerably affect the associations between environment characteristics and wellbeing measures. Although it is not easy to argue about the correctness of these measurements, what is evident is that they can tell us different things. While some methods can be used to determine the availability of certain environmental opportunities, others can provide us with insight into their relevance based on the actual exposure. Therefore, one might argue that the choice of spatial unit should be made based on the context and the contents of a study. Nevertheless, our findings suggest that over-simplistic and static residential units of analysis, such as administrative unit and home buffer, may not be suitable approaches for measuring the activities of individuals and, thus, capturing individual environmental exposure [23].

Furthermore, a growing body of research has used GPS data to capture the notions of activity spaces and the true environmental exposure of human behavior [22, 61, 62]. However, the resources, costs, and time that GPS data collection and acquisition demands often make it impractical for studies requiring large data sets. The activity spaces generated from a participatory mapping survey showed general consistency with the GPS activity spaces in this study and a study by Kestens et al. [32]. Thus, studying environmental exposure through participatory mapping seems accurate and precise compared to GPS but is less demanding, costly, and time consuming.

Some limitations of this research need to be acknowledged. This study examined only the association between the physical environment and perceived wellbeing, but other various background factors, such as jobs, family situations, and socioeconomic status, also play a role in individuals’ health. Previous studies have shown that demographic variables can mediate the relationships between health behavior and environmental variables [9]. Future research is warranted to investigate the multi-level influences of sociodemographic, built environment, and individual exposure variables on health. This study analyzed only a limited number of simple built environment variables in relation to perceived wellbeing. Using more complete and complex built environment variables may reveal more in-depth associations between the environment and health. Moreover, the comparisons made with GPS tracks are only based on a few participants. Future studies could benefit from a broader comparison based on a larger subsample of participants. In addition, the data used for this study were collected only from older adults aged 55–75. It would be interesting to see future studies exploring the presence of similar patterns in other age groups.

## Conclusions

The notion of the uncertain geographic context problem has been linked to the conventional spatial units of analysis used in multidisciplinary research fields studying the questions of health geographics. In this study, we applied two common residential and two novel activity space models as units of analysis to investigate whether there exist differences in the associations between built environment features and perceived individual health outcomes. According to the results of this study, different spatial units of analysis yield distinct results regarding the association between the built environment and health. Walkability index and the length of pedestrian and bicycle roads were found to positively correlate with perceived wellbeing measures only when the spatial context was captured with a home range model [33]. In contrast, a positive association between green spaces and perceived wellbeing was found when the individual exposure was assessed with IREM, which is a novel dynamic and people-based spatial model using data collected through online participatory mapping method to capture the notions of activity spaces [25].

This study investigated the challenges and opportunities of different spatial units of analysis and concludes that there are several suitable units of analysis that can be used to capture the human exposure. While one cannot simply argue about the correctness of a certain unit of analysis, what is evident is that they can tell us different things. The essential challenge that the broad field of health geographics needs to overcome is the comparability of different studies and results; using diverse, not standardized spatial units of analysis and measures makes comparisons hard if not impossible. However, as the results of this and previous studies show, researchers should not rely only on the easy and over-simplistic spatial units of analysis [18, 20, 22, 23, 32]. For a better assessment of contextual effects, researchers should more carefully consider different spatial units and evaluate their implications for the research outcomes.
